# Longitudinal Household Assessment of Respiratory Illness in Children and Parents During the COVID-19 Pandemic

**DOI:** 10.1001/jamanetworkopen.2022.37522

**Published:** 2022-10-20

**Authors:** Marieke L. A. de Hoog, Judith G. C. Sluiter-Post, Ilse Westerhof, Elandri Fourie, Valerie D. Heuvelman, Trisja T. Boom, Sjoerd M. Euser, Paul Badoux, Chantal Reusken, Louis J. Bont, Elisabeth A. M. Sanders, Vincent W. V. Jaddoe, Bjorn L. Herpers, Dirk Eggink, Joanne G. Wildenbeest, Liesbeth Duijts, Marlies A. van Houten, Patricia C. J. L. Bruijning-Verhagen

**Affiliations:** 1 Julius Centre for Health Sciences and Primary Care, Department of Epidemiology, University Medical Centre Utrecht, Utrecht, the Netherlands; 2 Spaarne Gasthuis Academy, Spaarne Gasthuis, Hoofddorp, the Netherlands; 3 Department of Pediatrics, Spaarne Gasthuis, Haarlem and Hoofddorp, the Netherlands; 4 Department of Pediatrics, Erasmus MC–Sophia Children’s Hospital, Erasmus University Medical Center, Rotterdam, the Netherlands; 5 Regional Public Health Laboratory Kennemerland, Haarlem, the Netherlands; 6 Centre for Infectious Disease Control, World Health Organization COVID-19 Reference Laboratory, National Institute for Public Health and the Environment, Bilthoven, the Netherlands; 7 Department of Pediatric Infectious Diseases and Immunology, Wilhelmina Children’s Hospital University Medical Center Utrecht, Utrecht, the Netherlands

## Abstract

**Importance:**

In the early COVID-19 pandemic, SARS-CoV-2 testing was only accessible and recommended for symptomatic persons or adults. This restriction hampered assessment of the true incidence of SARS-CoV-2 infection in children as well as detailed characterization of the SARS-CoV-2 disease spectrum and how this spectrum compared with that of other common respiratory illnesses.

**Objective:**

To estimate the community incidence of SARS-CoV-2 infection in children and parents and to assess the symptoms and symptom severity of respiratory illness episodes involving SARS-CoV-2–positive test results relative to those with SARS-CoV-2–negative test results.

**Design, Setting, and Participants:**

This cohort study randomly selected Dutch households with at least 1 child younger than 18 years. A total of 1209 children and adults from 307 households were prospectively followed up between August 25, 2020, and July 29, 2021, covering the second and third waves of the COVID-19 pandemic. Participation included SARS-CoV-2 screening at 4- to 6-week intervals during the first 23 weeks of participation (core study period; August 25, 2020, to July 29, 2021). Participants in all households finishing the core study before July 1, 2021, were invited to participate in the extended follow-up and to actively report respiratory symptoms using an interactive app until July 1, 2021. At new onset of respiratory symptoms or a SARS-CoV-2 positive test result, a household outbreak study was initiated, which included daily symptom recording, repeated polymerase chain reaction testing (nose-throat swabs and saliva and fecal samples), and SARS-CoV-2 antibody measurement (paired dried blood spots) in all household members. Outbreaks, households, and episodes of respiratory illness were described as positive or negative depending on SARS-CoV-2 test results. Data on participant race and ethnicity were not reported because they were not uniformly collected in the original cohorts and were therefore not representative or informative.

**Exposures:**

SARS-CoV-2–positive and SARS-CoV-2–negative respiratory illness episodes.

**Main Outcomes and Measures:**

Age-stratified incidence rates, symptoms, and symptom severity for SARS-CoV-2–positive and SARS-CoV-2–negative respiratory illness episodes.

**Results:**

Among 307 households including 1209 participants (638 female [52.8%]; 403 [33.3%] aged <12 years, 179 [14.8%] aged 12-17 years, and 627 [51.9%] aged ≥18 years), 183 household outbreaks of respiratory illness were observed during the core study and extended follow-up period, of which 63 (34.4%) were SARS-CoV-2 positive (59 outbreaks [32.2%] during the core study and 4 outbreaks [2.2%] during follow-up). SARS-CoV-2 incidence was similar across all ages (0.24/person-year [PY]; 95% CI, 0.21-0.28/PY). Overall, 33 of 134 confirmed SARS-CoV-2 episodes (24.6%) were asymptomatic. The incidence of SARS-CoV-2–negative respiratory illness episodes was highest in children younger than 12 years (0.94/PY; 95% CI, 0.89-0.97/PY). When comparing SARS-CoV-2–positive vs SARS-CoV-2–negative respiratory illness episodes in children younger than 12 years, no differences were observed in number of symptoms (median [IQR], 2 [2-4] for both groups), symptom severity (median [IQR] maximum symptom severity score, 6 [4-9] vs 7 [6-13]), or symptom duration (median [IQR], 6 [5-12] days vs 8 [4-13] days). However, among adults, SARS-CoV-2–positive episodes had a significantly higher number (median [IQR], 6 [4-8] vs 3 [2-4]), severity (median [IQR] maximum symptom severity score, 15 [9-19] vs 7 [6-11]), and duration (median [IQR] 13 [8-29] days vs 5 [3-11] days; *P* < .001 for all comparisons) of symptoms vs SARS-CoV-2–negative episodes.

**Conclusions and Relevance:**

In this cohort study, during the first pandemic year when mostly partial or full in-person learning occurred, the SARS-CoV-2 incidence rate in children was substantially higher than estimated from routine testing or seroprevalence data and was similar to that of adult household members. Unlike in unvaccinated adults, SARS-CoV-2 symptoms and symptom severity in children were similar to other common respiratory illnesses. These findings may prove useful when developing pediatric COVID-19 vaccine recommendations.

## Key Points

**Question:**

What is the incidence of SARS-CoV-2 infection in children detected when removing testing barriers, and how do symptoms and severity compare with the burden of other respiratory illnesses?

**Findings:**

In this cohort study of 1209 children and adults from 307 households in the Netherlands, when comparing the incidence (per person-year) of SARS-CoV-2 infection in children (0.22 at age <12 years and 0.21 at age 12-17 years) with that of adults (0.27), no significant difference was found. Unlike the SARS-CoV-2 disease burden in adults, the burden in children with SARS-CoV-2–positive respiratory illness episodes was similar to that of children with SARS-CoV-2–negative episodes.

**Meaning:**

This study found that SARS-CoV-2 symptoms in children were not more severe than symptoms from other common respiratory illnesses, which could be an important consideration in pediatric COVID-19 vaccine recommendations.

## Teaser

This cohort study examines the community incidence of SARS-CoV-2 infection among children and adults in the Netherlands and assesses the symptoms and symptom severity of respiratory illness episodes involving SARS-CoV-2 positivity vs SARS-CoV-2 negativity.

## Introduction

The COVID-19 pandemic remains a global health crisis, with more than 373 million confirmed cases reported globally as of February 1, 2022.^1^ Contrary to what has been observed for many other respiratory virus infections (eg, respiratory syncytial virus and influenza), children typically experience less severe disease with a SARS-CoV-2 infection compared with adults.^2^ It has been estimated that 20% to 50% of SARS-CoV-2 infections in individuals younger than 18 years occur without symptoms, approximately 20% of those with SARS-CoV-2 infections develop moderate illness, and fewer than 3% develop severe illness.^3,4^ Early in the pandemic, the role of children in the transmission of SARS-CoV-2 was thought to be small.^5,6^ However, this assumption was made at a time when schools were closed and COVID-19 testing facilities were only accessible to individuals experiencing symptoms related to SARS-CoV-2 infection. This situation hampered assessment of SARS-CoV-2 infections in children, and the true incidence may therefore have been underestimated. In addition, the ways in which the symptoms and incidence of SARS-CoV-2 infections compare with those of acute respiratory illness (ARI) due to other viruses in children has not been systematically quantified.

Through intensive longitudinal monitoring of households with children of different ages in the Netherlands, the Kids and SARS-CoV-2 Transmission and Disease (CoKids) study aimed to estimate the community incidence of symptomatic and asymptomatic SARS-CoV-2 infections and to assess the symptoms of individuals with SARS-CoV-2–positive ARI relative to those with SARS-CoV-2–negative ARI. The study was conducted between August 25, 2020, and July 29, 2021, during the period when the SARS-CoV-2 wild-type variant and the Alpha (B.1.1.7) variant of concern (VOC) circulated, which was replaced toward the end of June 2021 by the Delta (B.1.617.2) VOC. Children and adolescents had not yet been vaccinated against COVID-19. Vaccination of adults younger than 65 years was initiated during the final months of the study on April 27, 2021.

## Methods

### Study Population and Eligibility

This prospective cohort study was conducted in the Netherlands and was reviewed and approved by the Medical Ethical Committee Utrecht, the Medical Ethical Committee of the Vrije Universiteit University Medical Centre, and the Medical Ethical Committee of Erasmus Medical Centre. Written informed consent was obtained from all participating household members and/or their legal representatives. This study followed the Strengthening the Reporting of Observational Studies in Epidemiology (STROBE) reporting guideline for cohort studies.^7^

Eligible households participating in 3 existing Dutch birth cohort studies (the Respiratory Syncytial Virus Consortium in Europe [RESCEU] cohort [current age of children, 0-3 years],^8^ the Microbiome Utrecht Infant Study [MUIS] cohort [current age of children, 6-8 years],^9^ and the Generation R cohort [current age of children, 14-17 years]^10,11^), were approached for participation if they had 1 or more children aged 0 to 17 years. Household enrollment was conducted from August 25, 2020, through February 18, 2021. Detailed descriptions of the national COVID-19 prevention measures that were in place during the study period are described in eMethods in the Supplement.

### Study Procedures and Data Collection

The core study consisted of 23 weeks of longitudinal follow-up of each household for the occurrence of new-onset respiratory symptoms and/or fever, supplemented with repeated SARS-CoV-2 screening (midturbinate nose-throat swab [NTS] self-sampling) at 4- to 6-week intervals, irrespective of symptoms. Follow-up was temporarily intensified by means of an outbreak study that was initiated when (1) new-onset respiratory symptoms and/or fever in a household member developed, (2) a SARS-CoV-2 positive result on a screening test was received, or (3) a reported positive test result at an external testing site was received. Outbreaks, households, and episodes of respiratory illness were described as positive or negative depending on SARS-CoV-2 test results. The outbreak study included daily symptom diaries and repeated NTS, saliva and fecal self-sampling, and serological testing (dried blood spot by self–finger prick) for all household members. For study procedures and data collection, we used a custom-made study app. Detailed descriptions of the study procedures and data collection and the full outbreak sampling protocols for SARS-CoV-2–positive and SARS-CoV-2–negative outbreaks are described in eMethods and eTable 1 in the Supplement. To limit the burden of study procedures for participants, follow-up was ended before the study completion date after a household had contributed to 2 outbreak studies or after a confirmed SARS-CoV-2–positive outbreak. Data on participant race and ethnicity were not reported because they were not uniformly collected in the original cohorts and were therefore not representative or informative.

The core study was extended beyond 23 weeks. Participants in all households finishing the core study before July 1, 2021, were invited to participate in the extended follow-up and to actively report respiratory symptoms using the interactive app until July 1, 2021. The extended follow-up no longer included repeated SARS-CoV-2 screening, and the outbreak study sampling protocol was only initiated for confirmed SARS-CoV-2 infection in the household.

### Laboratory Analyses

Nose-throat swabs and saliva and fecal samples were tested for the presence of SARS-CoV-2 by a reverse transcription–polymerase chain reaction (RT-PCR) test, as described elsewhere.^12^ Specimens with a cycle threshold (or crossing point) value of less than or equal to 40 were defined as SARS-CoV-2 positive. To assess the presence of antibodies against SARS-CoV-2, dried blood spot specimens were tested in a final dilution of 1:40 using a multiplex protein microarray for immunoglobin G antibodies reactive with the SARS-CoV-2 trimeric spike glycoprotein and nucleocapsid antigens, as described elsewhere.^13^ A signal exceeding 45 000 fluorescent units was considered positive. To assess which SARS-CoV-2 types were prevalent in the SARS-CoV-2–positive households, all positive RT-PCR NTS results were sequenced, as described previously.^14^ Details of the laboratory analysis are available in eMethods in the Supplement.

### Case Definitions

Confirmed SARS-CoV-2 infection was defined as (1) a positive RT-PCR result from NTS or saliva or fecal samples or a documented positive SARS-CoV-2 result on an antigen or PCR test from an official external source (ie, a municipal testing facility), (2) a SARS-CoV-2 negative serological test result at the start of an outbreak period and a positive serological test result at the end of an outbreak period (ie, seroconversion), or (3) a positive serological test result at the start of an outbreak period and respiratory symptoms in the previous 2 weeks that were not previously reported to the study team. Probable SARS-CoV-2 infection was defined as an episode of respiratory symptoms (at least 1 day of fever or 2 consecutive days with at least 1 respiratory symptom, including nasal congestion and/or runny nose, cough, sore throat, or shortness of breath) without confirmation by RT-PCR or serological testing but in the presence of 1 household member with an RT-PCR–confirmed SARS-CoV-2 infection. A SARS-CoV-2–negative respiratory illness episode was defined as the presence of respiratory symptoms when all samples had negative results for SARS-CoV-2 during the outbreak period, both for the participant and all household members.

An ARI was classified based on daily symptom reports if there was either (1) new onset of fever or (2) 2 consecutive days with at least 1 respiratory symptom (cough, sore throat, cold, or dyspnea) and 1 systemic symptom (headache, muscle ache, cold shivers, or fatigue) or 2 respiratory symptoms regardless of the outcome of a SARS-CoV-2 test. For ARI episodes, we collected additional data on severity of symptoms daily using a 5-point investigator-designed scale (with higher severity scores indicating greater symptom severity), and a disease questionnaire detailing health care visits and medication use was completed at the end of the episode.

### Statistical Analysis

The SARS-CoV-2 incidence rate by age category (<12 years, 12-17 years, and ≥18 years) was calculated by dividing the number of SARS-CoV-2 infections by the person-time of observation. We calculated rates using only confirmed SARS-CoV-2 infections and rates including both confirmed and probable SARS-CoV-2 infections. Similarly, we calculated the incidence rate of episodes of SARS-CoV-2–negative respiratory illness for different age groups. The incidence calculations were based on results of the core study period because, during the extended follow-up period, SARS-CoV-2–negative outbreaks were no longer studied. In addition, no SARS-CoV-2 screenings were performed during the extended follow-up period, and outbreak entry was only based on a positive SARS-CoV-2 NTS test result from the index case, reducing the overall sensitivity of the protocol to detect SARS-CoV-2 infections. Next, we calculated the SARS-CoV-2 attack rate (calculated as the number of participants with a SARS-CoV-2 infection divided by the number of household members involved in a SARS-CoV-2 outbreak) and described symptom frequency and severity by comparing SARS-CoV-2–positive and SARS-CoV-2–negative respiratory illness episodes. For these analyses, all data collected during the entire study period were used. We used rate ratio tests to compare incidence rates and attack rates across age groups. Symptoms were tested for differences by age group and SARS-CoV-2 status. The χ^2^ test was used to assess symptom frequency, and the Mann-Whitney *U* test was used to assess symptom duration, number of symptoms, and maximum symptom severity score.

Two-tailed *P* < .05 was considered statistically significant. Statistical analyses were performed using R software, version 4.0.3 (R Foundation for Statistical Computing).

## Results

### Study Population and Households

From August 2020 until February 2021, a total of 307 households including 1209 participants (638 female [52.8%] and 571 male [47.2%]) between ages 0 and 69 years were enrolled in the prospective household study (Table 1). Overall, 157 families were recruited from the RESCEU cohort, 47 families were recruited from the MUIS cohort, and 103 families were recruited from the Generation R cohort. In total, 403 children (33.3%) younger than 12 years, 179 children (14.8%) aged 12 to 17 years, and 627 adults (51.9%) were enrolled. Of 627 adults, 205 (32.7%) were unvaccinated for the entire duration of the study, 74 (11.8%) received at least 1 vaccine dose during the core study, and an additional 287 (45.8%) received at least 1 vaccine dose during the period of extended follow-up. For 58 adults (9.3%), the vaccination status was unknown. None of the adults with a SARS-CoV-2 infection were vaccinated at the time of infection. No children younger than 18 years were vaccinated during the study period.

**Table 1.** Baseline Characteristics of Study Population

Characteristic	Households or household members, No./total No. (%)		
	Total	Confirmed SARS-CoV-2 positive	SARS-CoV-2 negative
Total households, No.	307	63	244
Total household members, No.	1209	252	957
Total household outbreaks			
Core study	179/183 (97.8)	59/63 (93.7)	120/120 (100)
Extended follow-up	4/183 (2.2)	4/63 (6.3)	0
Total persons in outbreaks			
Core study	710/727 (97.7)^a^	235/252 (93.3)	475/475 (100)
Extended follow-up	17/727 (2.3)^a^	17/252 (6.7)	0
Household size, No. of members			
2	5/307 (1.6)	2/63 (3.2)	3/244 (1.2)
3	72/307 (23.5)	12/63 (19.0)	60/244 (24.6)
4	159/307 (51.8)	34/63 (54.0)	125/244 (51.2)
5	58/307 (18.9)	11/63 (17.5)	47/244 (19.3)
>5	13/307 (4.2)	4/63 (6.3)	9/244 (3.7)
Sex			
Female	638/1209 (52.8)	130/252 (51.6)	508/957 (53.1)
Male	571/1209 (47.2)	122/252 (48.4)	449/957 (46.9)
Age at inclusion, y			
<12	403/1209 (33.3)	89/252 (35.3)	314/957 (32.8)
12-17	179/1209 (14.8)	37/252 (14.7)	142/957 (14.8)
≥18	627/1209 (51.9)	126/252 (50.0)	501/957 (52.4)

^a^ Persons were counted twice if they participated in more than 1 SARS-CoV-2–positive and/or SARS-CoV-2–negative respiratory outbreak.

A total of 183 respiratory outbreaks in 150 households were detected during the core study period, of which 59 (32.2%) were SARS-CoV-2 positive and 120 (65.6%) were SARS-CoV-2 negative. An additional 4 SARS-CoV-2–positive outbreaks (2.2%) were detected during the extended follow-up period, resulting in 63 SARS-CoV-2–positive outbreaks (34.4%) during the core study and extended follow-up. Thirty households had 2 or more outbreaks of respiratory symptoms, of which 12 had both a SARS-CoV-2–positive and a SARS-CoV-2–negative outbreak. In total, 157 of 307 households (51.1%) did not report any respiratory symptoms or SARS-CoV-2 infection during the entire follow-up. In households with vs without SARS-CoV-2 infection, household size (eg, 4 members: 34 of 63 households [54.0%] vs 125 of 244 households [51.2%]), sex (eg, 130 of 252 female individuals [51.6%] vs 508 of 957 female individuals [53.1%]), and age distribution (eg, <12 years: 89 of 252 individuals [35.3%] vs 314 of 957 individuals [32.8%]) were similar (Table 1). At the start of an outbreak, 25 of 230 total participants (10.9%) had a positive serological result during a SARS-Cov-2–positive outbreak, and 6 of 350 participants (1.7%) had a positive serological result during a SARS-CoV-2–negative outbreak. A total of 67 of the 69 RT-PCR–positive NTS results (97.1%) were successfully sequenced, containing data from 34 SARS-CoV-2–positive household outbreaks involving 65 individuals. Samples from 37 of those individuals (56.9%) contained RNA of the ancestral strain, and 28 (43.1%) contained RNA of the Alpha VOC. Sample completeness varied substantially by time point and sample type. For example, convalescent serological results were only available for 152 of 252 participants (60.3%) in SARS-CoV-2–positive outbreaks and 221 of 475 participants (46.5%) in SARS-CoV-2–negative outbreaks. Additional details are available in eTable 2 in the Supplement.

### Incidence of SARS-CoV-2–Positive and SARS-CoV-2–Negative Respiratory Illness Episodes

The incidence rates of SARS-CoV-2–positive and SARS-CoV-2–negative respiratory illness episodes in the different age groups during the core study are shown in Table 2. Within the 59 SARS-CoV-2 household outbreaks during the core study, a total of 123 confirmed SARS-CoV-2 infections were detected during 26 883 person-weeks of follow-up. An additional 60 participants developed symptoms during a SARS-CoV-2 outbreak in the household that could not be confirmed by RT-PCR or serological testing but were considered probable cases. The overall incidence of SARS-CoV-2 was 0.24/person-year (PY; 95% CI, 0.21-0.28/PY) for confirmed SARS-CoV-2 only and 0.36/PY (95% CI, 0.31-0.40/PY) for confirmed and probable SARS-CoV-2 cases combined. Overall, no differences in SARS-CoV-2 incidence rates were found between the different age groups (eg, confirmed SARS-CoV-2 infections for age <12 years vs 12-17 years: 0.22/PY [95% CI, 0.16-0.29/PY] vs 0.21/PY [95% CI, 0.13-0.32/PY]; *P* = .95). SARS-CoV-2–negative respiratory illness episodes occurred most frequently in children younger than 12 years (0.94/PY [95% CI, 0.89-0.97/PY] vs 0.25/PY [95% CI, 0.16-0.37/PY] in children aged 12-17 years and 0.58/PY [95% CI, 0.52-0.64/PY] in adults; *P* < .001 for all comparisons). Of all respiratory illness episodes reported in children younger than 12 years, 37 of 231 (16.0%) were confirmed SARS-CoV-2 infections, and 69 of 231 (29.9%) were confirmed or probable SARS-CoV-2 infections . Among children aged 12 to 17 years, 16 of 43 respiratory illness episodes (37.2%) were confirmed SARS-CoV-2 infections, and 24 of 43 (55.8%) were confirmed or probable SARS-CoV-2 infections. Among adults, 72 of 246 respiratory illness episodes (29.3%) were confirmed SARS-CoV-2 infections, and 91 of 246 (37.0%) were confirmed or probable SARS-CoV-2 infections.

**Table 2.** Incidence of SARS-CoV-2–Positive and SARS-CoV-2–Negative Respiratory Illness Episodes by Age Group

Age group, y	Follow-up time, wk	Confirmed SARS-CoV-2 positive		Confirmed SARS-CoV-2 positive or probable		SARS-CoV-2 negative	
		Episodes, No.	Incidence/PY (95% CI)^a^	Episodes, No.	Incidence/PY (95% CI)^a^	Episodes, No.	Incidence/PY (95% CI)^a^
<12	8985	37	0.22 (0.16-0.29)	69	0.40 (0.33-0.48)	162	0.94 (0.89-0.97)
12-17	3965	16	0.21 (0.13-0.32)	24	0.30 (0.21-0.42)	19	0.25 (0.16-0.37)
≥18	13 933	72	0.27 (0.22-0.33)	91	0.34 (0.29-0.40)	155	0.58 (0.52-0.64)
All	26 883	123	0.24 (0.21-0.28)	183	0.36 (0.31-0.40)	329	0.65 (0.61-0.69)

Abbreviation: PY, person-year.

^a^ Incidence rates were based on household outbreaks occurring during the core study period.

Among the 134 confirmed SARS-CoV-2 infections detected during the entire study period (including the extended follow-up period), 33 cases (24.6%) were asymptomatic. The proportion of asymptomatic SARS-CoV-2 infections did not differ between age groups (14 of 89 cases [15.7%] for age <12 years, 5 of 37 cases [13.5%] for age 12-17 years, and 14 of 126 cases [11.1%] for adults; *P* = .28). The overall attack rate for SARS-CoV-2 household outbreaks was 53.2% (95% CI, 46.8%-59.4%) when considering only confirmed SARS-CoV-2 cases and 77.4% (95% CI, 71.6%-82.3%) when including both confirmed and probable cases, and attack rates were similar across ages (Table 3).

**Table 3.** Confirmed and Probable SARS-CoV-2 Cases and Attack Rate by Age Group in 63 SARS-CoV-2 Household Outbreaks

Age group, y	Participants in outbreak, No.	Cases, No. (%)				Attack rate, No. (%) [95% CI]	
		Confirmed SARS-CoV-2 positive		Confirmed SARS-CoV-2 probable	SARS-CoV-2 negative^a^	Confirmed SARS-CoV-2 positive	Confirmed SARS-CoV-2 positive or probable
		Asymptomatic	Symptomatic				
<12	89	14 (15.7)	29 (32.6)	33 (37.0)	13 (14.6)	43 (48.3) [37.7-59.1]	76 (85.4) [76.0-91.7]
12-17	37	5 (13.5)	11 (29.7)	7 (18.9)	14 (37.8)	16 (43.2) [27.5-60.4]	23 (62.2) [44.8-77.1]
≥18	126	14 (11.1)	61 (48.4)	21 (16.7)	30 (23.8)	75 (59.5) [50.4-68.1]	96 (76.2) [67.6-83.1]
All	252	33 (13.1)	101 (40.1)	61 (24.2)	57 (22.6)	134 (53.2) [46.8-59.4]	195 (77.4) [71.6-82.3]

^a^ Includes participants who were asymptomatic and had negative results for SARS-CoV-2 infection in all samples tested during the outbreak.

### Characteristics of SARS-CoV-2–Positive and SARS-CoV-2–Negative Respiratory Illness Episodes

The symptoms and symptom severity of SARS-CoV-2–positive and SARS-CoV-2–negative respiratory illness episodes stratified for children and adults are shown in the Figure, Table 4, and eFigure 1 in the Supplement. In children, no clear distinction could be made between SARS-CoV-2–positive vs SARS-CoV-2–negative respiratory illness episodes based on the reported symptoms (Figure panels A and C; eFigure 1A and 1C in the Supplement). Children younger than 12 years reported symptoms for a median (IQR) duration of 6 (5-12) days with a median (IQR) maximum symptom severity score of 6 (4-9) for SARS-CoV-2–positive respiratory illness episodes and a median (IQR) duration of 8 (4-13) days with a median (IQR) maximum symptom severity score of 7 (6-13) for SARS-CoV-2–negative respiratory illness episodes. The median (IQR) number of reported symptoms was 2 (2-4) in both SARS-CoV-2–positive and SARS-CoV-2–negative groups. In adults, generally more respiratory symptoms with greater severity were reported for a longer duration during a SARS-CoV-2–positive respiratory illness episode compared with a SARS-CoV-2–negative episode (median [IQR] number of symptoms: 6 [4-8] vs 3 [2-4]; median [IQR] maximum symptom severity score: 15 [9-19] vs 7 [6-11]; median [IQR] duration of symptoms: 13 [8-29] days vs 5 [3-11] days; *P* < .001 for all comparisons) (Table 4; Figure panels B and D; eFigure 1B and 1D in the Supplement).

**Figure.**
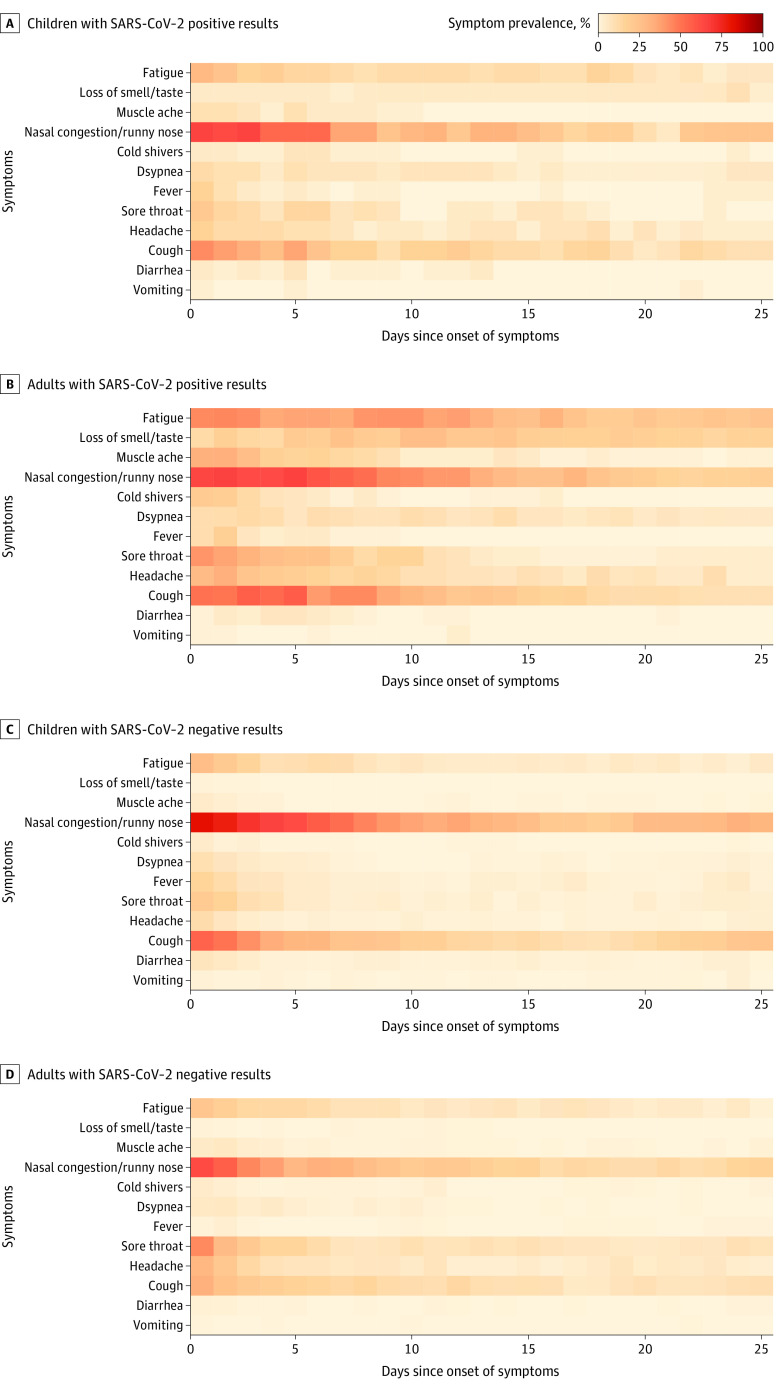
Heat Map of Individual Symptoms Up to Day 25 of SARS-CoV-2 Positive and Negative Respiratory Illness Episodes in Children and Adults Symptom prevalence refers to the proportion of participants reporting a particular symptom on a given day (calculated as the number of participants reporting a particular symptom divided by the total number of participants completing a diary entry on a given day).

**Table 4.** Disease Characteristics of SARS-CoV-2–Positive and SARS-CoV-2–Negative Respiratory Illness Episodes by Age Group

Characteristic	Median (IQR)					
	Symptomatic confirmed SARS-CoV-2 positive			SARS-CoV-2 negative		
	Age <12 y (n = 29)	Age 12-17 y (n = 11)	Age ≥18 y (n = 61)	Age <12 y (n = 162)	Age 12-17 y (n = 19)	Age ≥18 y (n = 155)
Days with symptoms	6 (5-12)	7 (4-28)	13 (8-29)	8 (4-13)	6 (5-11.5)	5 (3-11)
No. of symptoms	2 (2-4)	5 (2-8)	6 (4-8)	2 (2-4)	4 (3-6)	3 (2-4)
Maximum symptom severity score^a^	6 (4-9)	13 (9-21)	15 (9-19)	7 (6-13)	10 (6-13)	7 (6-11)

^a^ Symptom severity score was only requested for participants with episodes meeting the case definition of acute respiratory illness.

Nasal congestion and/or runny nose with or without cough or fatigue were the 3 most prevalent symptom combinations for both SARS-CoV-2–positive and SARS-CoV-2–negative respiratory illness episodes in both adults (nasal congestion and/or runny nose: 58 of 61 adults [95.1%] with SARS-CoV-2 infection vs 120 of 155 adults [77.4%] without SARS-CoV-2 infection; cough: 49 of 61 adults [80.3%] with SARS-CoV-2 infection vs 80 of 155 adults [51.6%] without SARS-CoV-2 infection; fatigue: 46 of 61 adults [75.4%] with SARS-CoV-2 infection vs 58 of 155 adults [37.4%] without SARS-CoV-2 infection) and children (nasal congestion and/or runny nose: 34 of 40 children [85.0%] with SARS-CoV-2 infection vs 166 of 181 children [91.7%] without SARS-CoV-2 infection; cough: 25 of 40 children [62.5%] with SARS-CoV-2 infection vs 119 of 181 children [65.7%] without SARS-CoV-2 infection; fatigue: 17 of 40 children [42.5%] with SARS-CoV-2 infection vs 69 of 181 children [38.1%] without SARS-CoV-2 infection) (eFigure 1 in the Supplement). Among adults with SARS-CoV-2–positive episodes, fatigue frequently persisted for 2 weeks or more (Figure panel B). Loss of smell and/or taste was reported by 25 of 61 adults (41.0%) with SARS-CoV-2–positive episodes vs 6 of 155 adults (3.9%) with SARS-CoV-2–negative episodes. In children, this symptom was uncommon in both groups (3 of 40 children [7.5%] with SARS-CoV-2–positive episodes vs 3 of 181 children [1.7%] with SARS-CoV-2–negative episodes). In SARS-CoV-2–positive episodes, adults compared with children younger than 12 years also experienced a higher number of symptoms (median [IQR], 6 [4-8] vs 2 [2-4]; *P* < .001), a longer duration of symptoms (median [IQR], 13 [8-29] days vs 6 [5-12] days; *P* < .001), and higher severity of symptoms (median [IQR] maximum severity score, 15 [9-19] vs 6 [4-9]; *P* < .001) (Table 4; eFigure 2 in the Supplement).

## Discussion

In this cohort study, which was conducted during the second and third waves of the COVID-19 pandemic when adults and children in the Netherlands were largely unvaccinated and schools operated at variable levels of in-person learning, we observed that SARS-CoV-2 infection rates did not significantly differ between children and adults. When infected with SARS-CoV-2, younger age groups experienced fewer and milder disease symptoms compared with adults. In children, SARS-CoV-2 symptoms and symptom severity among those with SARS-CoV-2–positive respiratory illness could not be distinguished from those with SARS-CoV-2–negative respiratory illness. To our knowledge, this study is the first contemporary assessment of symptoms and disease burden for both SARS-CoV-2–positive and SARS-CoV-2–negative respiratory illnesses in children and adults.

In the early days of the pandemic, there was a general notion that children were less likely to get infected with SARS-CoV-2.^5,6^ However, this assumption was largely based on observations from settings in which schools were closed and SARS-CoV-2 testing was only accessible and recommended for symptomatic persons. Our study found that, with an intensive testing protocol that made no distinction in symptom severity, types of symptoms, or age, the measured incidence among children and their parents was similar and that, once SARS-CoV-2 entered the family, the attack rates did not vary significantly between the studied age groups. These findings suggest that differences in susceptibility to infection by age may be smaller than previously thought. Our findings are consistent with the SARS-CoV-2 incidence rates found in a US study in which 1236 participants from 310 households with children were self-sampled weekly for SARS-CoV-2 infection between September 2020 and April 2021.^3^ Alternatively, the differences may have narrowed with the emergence of the more infectious Alpha variant that became dominant during our study period.

Dutch serological data revealed that, in June 2021, 17% to 20% of children younger than 12 years had antibodies against SARS-CoV-2.^15^ This incidence rate is lower than the incidence rates of 0.22 confirmed SARS-CoV-2 infections per PY and 0.40 confirmed or probable SARS-CoV-2 infections per PY found in our study, which suggests that during the pandemic period up to the emergence of the Omicron (B.1.1.529) variant in December 2021, at least one-third and probably up to two-thirds of children younger than 12 years in the Netherlands had already experienced an episode of SARS-CoV-2 infection. This proportion has likely further increased due to the substantial wave of Omicron infections in the population.^16^ It is therefore likely that few children are still fully naive to SARS-CoV-2 at this stage of the pandemic. This finding should be considered in pediatric COVID-19 vaccine recommendations. In children with previous exposure to SARS-CoV-2, reduced dosing or single-dose schedules could be sufficient to protect against severe disease outcomes.

Our study also provided a granular and direct comparison of the symptoms of SARS-CoV-2–positive and SARS-CoV-2–negative respiratory illness episodes in the community setting. We observed that the symptoms and severity of disease were similar for SARS-CoV-2–positive vs SARS-CoV-2–negative respiratory illness episodes in children, especially in children younger than 12 years, whereas among adults, SARS-CoV-2–positive episodes had a greater symptom burden and longer disease course compared with SARS-CoV-2–negative episodes. Among children, the most common symptom combinations were runny nose and/or nasal congestion with or without cough and fatigue for both SARS-CoV-2–positive and SARS-CoV-2–negative episodes. This finding confirms that, in most children, it is not possible to distinguish SARS-CoV-2 illness from other respiratory illnesses based on symptoms. In adults, loss of smell and/or taste is a discriminative symptom for SARS-CoV-2 infection and was present in 41.0% of those with positive SARS-CoV-2 test results vs 3.9% of those with negative test results; however, in children, this symptom was uncommon in both groups (7.5% with positive SARS-CoV-2 results vs 1.7% with negative results). However, among children, there might have been underreporting of symptoms that were difficult to identify by the children or their parents.

Of note, recent data have suggested that loss of smell and/or taste may be less common with Omicron infection.^17,18^ Our results also further confirmed that the disease burden from SARS-CoV-2 infection in children is generally mild^19,20,21,22^ and comparable with that of other respiratory illnesses. Notably, influenza virus and respiratory syncytial virus are known to be associated with more severe respiratory disease in young children,^23,24,25,26,27^ but these viruses were not circulating during the period when this study was conducted, presumably as a result of the COVID-19 nonpharmaceutical control interventions. This deviation in seasonal pattern may have lowered the mean disease burden of SARS-CoV-2–negative respiratory illness episodes in children in our study compared with normal seasonal patterns. In addition, during our study, only a small percentage of the adults were vaccinated; therefore, our findings regarding the SARS-CoV-2 disease burden in adults cannot be extrapolated to vaccinated individuals.

### Limitations

This study has several limitations. First, despite the extensive sampling protocol, it is possible that SARS-CoV-2 infections were underdetected to some extent due to missing outbreak samples, asymptomatic infections occurring between screenings, and the fact that study participation for some households ended before the study completion date. Sample completeness was high for the initial outbreak samples but decreased thereafter, and convalescent serological results were only available for 60.3% and 46.5% of participants in SARS-CoV-2–positive and SARS-CoV-2–negative outbreaks, respectively. Therefore, the incidence and attack rates reported in this study should be interpreted as minimum rather than mean values. Similarly, the true proportion of asymptomatic infections could be somewhat higher than estimated in this study.

Second, this study was performed before the Omicron variant emerged. It has been reported that Omicron infection is associated with less severe disease and possibly higher transmissibility.^28,29^ Therefore, symptoms of SARS-CoV-2 infections in unvaccinated children may now have changed, with a further reduction in severity compared with our observations.

Third, this study was conducted in the Netherlands. Incidence rates during the same period may have been different in other countries and settings depending on the level of epidemic control and measures such as school closures. The SARS-CoV-2 incidence estimates should be interpreted in the context of the prevailing COVID-19 situation at the time and location of the study.

## Conclusions

In this cohort study, the incidence rate of SARS-CoV-2 infections in children was similar to that of adults during a pandemic period in which mostly partial or full in-person learning occurred. This rate contrasts with earlier findings revealing that children may be less susceptible to becoming infected with SARS-CoV-2 . The results of this study confirmed that SARS-CoV-2 symptom severity was substantially lower in children compared with adults if both were unvaccinated. Symptoms and symptom severity of SARS-CoV-2 infection in children were similar to those of respiratory illnesses from other causes that occurred during the same period. These findings should be considered when developing pediatric COVID-19 vaccine recommendations.

## Notes

Supplement. **eMethods.** National COVID-19 Prevention Measures During the Study Period, Study Procedures and Data Collection, and Laboratory Analyses **eTable 1.** Detailed Study Sampling Scheme for All Participants Enrolled **eTable 2.** Proportion of Sample Completeness of SARS-CoV-2–Positive and SARS-CoV-2–Negative Respiratory Outbreaks **eFigure 1.** Most Common Symptom Combinations per Episode of SARS-CoV-2–Positive and SARS-CoV-2–Negative Respiratory Illness Episodes in Children (Aged <18 Years) and Adults **eFigure 2.** Symptom Severity Score Since Onset of ARI Symptoms for SARS-CoV-2–Positive and SARS-CoV-2–Negative Respiratory Illness Episodes
eReferences
