# Supplementary material for: Longitudinal Household Assessment of Respiratory Illness in Children and Parents During the COVID-19 Pandemic
Source: JAMA Netw Open. 2022 Oct 20;5(10):e2237522. doi: 10.1001/jamanetworkopen.2022.37522 (PMC9585428; doi:10.1001/jamanetworkopen.2022.37522)
Supplement: Supplement. — eMethods. National COVID-19 Prevention Measures During the Study Period, Study Procedures and Data Collection, and Laboratory Analyses eTable 1. Detailed Study Sampling Scheme for All Participants Enrolled eTable 2. Proportion of Sample Completeness of SARS-CoV-2–Positive and SARS-CoV-2–Negative Respiratory Outbreaks eFigure 1. Most Common Symptom Combinations per Episode of SARS-CoV-2–Positive and SARS-CoV-2–Negative Respiratory Illness Episodes in Children (Aged <18 Years) and Adults eFigure 2. Symptom Severity Score Since Onset of ARI Symptoms for SARS-CoV-2–Positive and SARS-CoV-2–Negative Respiratory Illness Episodes eReferences [file jamanetwopen-e2237522-s001.pdf]

Supplementary Online Content

de Hoog MLA, Sluiter-Post JGC, Westerhof I, et al. Longitudinal household assessment of respiratory illness in children and parents during the COVID-19 pandemic. *JAMA Netw Open*. 2022;5(10):e2237522. doi:10.1001/jamanetworkopen.2022.37522

eMethods. National COVID-19 Prevention Measures During the Study Period, Study Procedures and Data Collection, and Laboratory Analyses

eTable 1. Detailed Study Sampling Scheme for All Participants Enrolled

eTable 2. Proportion of Sample Completeness of SARS-CoV-2–Positive and SARS-CoV-2–Negative Respiratory Outbreaks

eFigure 1. Most Common Symptom Combinations per Episode of SARS-CoV-2–Positive and SARS-CoV-2–Negative Respiratory Illness Episodes in Children (Aged <18 Years) and Adults

eFigure 2. Symptom Severity Score Since Onset of ARI Symptoms for SARS-CoV-2–Positive and SARS-CoV-2–Negative Respiratory Illness Episodes

eReferences

This supplementary material has been provided by the authors to give readers additional information about their work.

© 2022 de Hoog MLA et al. *JAMA Network Open*.

eMethods. National COVID-19 Prevention Measures During the Study Period, Study Procedures and Data Collection, and Laboratory Analyses

National COVID-19 prevention measures during the study period

During the study period, the extent of national COVID-19 prevention measures varied. Throughout the study stay-at-home orders for persons with respiratory complaints or fever and physical distancing orders (>1.5 meters) were in place as well as restrictions on large social gatherings. From October 2020 until March 2021, bars and restaurants were (partially) closed and sports activities were only allowed outdoors. Schools and daycares were operating under full occupancy until mid-December 2020, when they were closed for in-person learning and remained so throughout January 2021. In early February 2021, elementary schools and daycare reopened at half occupancy followed by secondary schools in March 2021. All schools operated at full occupancy again from May 2021 onwards. COVID-19 testing was available and recommended at municipal health testing facilities for persons with symptoms throughout the study, and for asymptomatic, exposed individuals from December 2020 onwards. COVID-19 antigen self-tests became widely available in March 2021.

Study procedures and data collection

Household enrolment ran from August 2020 through February 2021. At study enrolment, a baseline questionnaire was completed regarding household characteristics, and medical and demographic characteristics of each household member. During a home visit, research staff provided instructions on self-sampling of a combined mid turbinate nose-throat swab (NTS) and saliva sampling by means of Oracol® sponges. Self-sampling was further supported by instruction videos and leaflets.

Households were instructed to actively report new onset of respiratory symptoms or fever in any of the household members throughout follow-up. In addition, SARS-CoV-2 screening at 4-6 weeks interval was performed on self-collected NTS during the first 23 weeks of follow-up (“core study”), that were PCR tested within 24 hrs. An outbreak study was launched every time that at least one household member reported new onset of respiratory symptoms or fever, or if a screening test (in core study) or external test yielded a SARS-CoV-2 positive result. The core study was extended (“extended follow-up”) until July 1st 2021 to allow for continued follow-up and no longer included repeated SARS-CoV-2 screening and the outbreak study sampling protocol was only initiated for confirmed SARS-CoV-2 infection in the household.

At the start of an outbreak, NTS and saliva were collected within 48hrs of symptom onset in the index case from all household members, irrespective of symptoms. All NTS samples were PCR tested within 24h for presence of

© 2022 de Hoog MLA et al. *JAMA Network Open*.

SARS-CoV-2 virus. Follow-up household sampling schemes were stratified based on presence of SARS-CoV-2 in the initial NTS outbreak samples. The full outbreak sampling protocols for SARS-CoV-2 positive and negative outbreaks are summarized in supplement Table 1. This includes: 1) Dried Blood Spot (DBS) by self-finger-prick at the start of the outbreak study and ten days after completion of the outbreak period (convalescent sample); 2) additional NTS and saliva for symptomatic subjects throughout the outbreak period as well as 3) frequent saliva and fecal sampling for SARS-CoV-2 positive outbreaks.

In the extended follow-up, the outbreak study entry criteria were slightly adapted; immediate NTS results were only available for the person(s) who reported respiratory symptoms (index case). If SARS-CoV-2 positive, the outbreak study was initiated.

All samples were temporarily stored in the participant's home freezer, collected by research assistants from home, and subsequently transported to the laboratory on dry ice and stored at -80 degrees Celsius (°C) until further analysis.

Monitoring of respiratory symptoms was temporarily intensified using daily symptom diaries for each household member, supplemented with daily symptom severity scores and disease questionnaires for those episodes meeting the case definition for Acute Respiratory Illness (ARI). The following symptoms were included in the diary; nasal congestion/runny nose, cough, headaches, sore throat, fever, shortness of breath, cold shivers, muscle aches, loss of taste or smell, fatigue, vomiting and diarrhea. The outbreak study lasted at least 21 days or until 21 days after the last ARI onset in a household member.

For study procedures and data collection we used a custom made study app (COVapp), compatible with Apple and Android systems, developed by the University Medical Center Utrecht (UMCU) in collaboration with YourResearch Holding BV. The study app was used to collect four types of self-reported data: (1) baseline questionnaire data, (2) reporting of new onset of respiratory symptoms or fever, (3) registration of all virological and serological specimens collected through participant self-sampling, (4) symptom diaries and disease questionnaires in case of an outbreak. All data entered in the App by the participants were stored in an online secured database and could be accessed and navigated in real-time by study personnel using an online portal with authorized login.

© 2022 de Hoog MLA et al. *JAMA Network Open*.

Laboratory analyses

NTS were analysed using the SARS-CoV-2 RT-PCR based on the presence of the E-gene, [1] which was carried out after a lysis step of the samples. Crossing point (Cp)-values were calculated on Lightcycler 480 1.5.1 software (Roche diagnostics, Basel, Switzerland). This was done by the Regional Public Health Laboratory Kennemerland, Haarlem, The Netherlands.

SARS-CoV-2 presence and viral loads in saliva and fecal samples were analysed by the laboratory of the National Institute for Public Health and the Environment (RIVM), Bilthoven, the Netherlands. The Oracol® sponges were processed according to the manufacturer's instructions and saliva of both sponges was pooled before further analysis. Total nucleic acid was extracted from 200 µl saliva using MagNApure 96 (MP96) with total nucleic acid kit small volume (Roche). Equine arteritis virus (EAV) was added to the 275 µl lysisbuffer as internal control and yeast tRNA was added as stabilizer. Total nucleic acid was eluted in 50 µl Tris EDTA buffer. Of the feces specimens a 5% suspension was made in MEM with Hanks' salts and penicillin and streptomycin, vortex for 15 seconds and 1 minute centrifuged at 16,000 Relative Centrifugal Force. Two-hundred µl supernatant was mixed with 275 µl MP96 lysis buffer including equine arteritis virus (EAV) internal control and yeast tRNA stabilizer.RT-qPCR was performed on 5 µl total nucleic acid using TaqMan® Fast Virus 1-Step Master Mix (Thermo Fisher) on Roche LC480 II thermal cycler with SARS-like beta coronavirus (Sarbeco) specific E-gene primers and RdRp probe and EAV as described.[1]

NTS, saliva and fecal specimens with a cycle threshold (Cp-/Ct-values) less than or equal to 40 were defined as SARS-CoV-2 positive.

DBS were transported on dry ice to the laboratory, where they were stored at -80˚C. Two bloodspots of 3/16" of serum were punched out of the filter paper and were incubated in 200 µl Blotto-blockingbuffer containing 5% Surfact-Amps 20 (ThermoFisher Scientific Inc., Rockford, USA) overnight at 4°C to release serum from DBS for a test dilution of approximately 1 in 40. 90 µl of the eluted serum was used and subsequently tested for the presence of IgG antibodies reactive with the SARS-CoV-2 spike trimer, S1 and SARS-CoV-2 N antigens in a protein microarray, essentially as described previously.[2] Signal exceeding 45,000 fluorescent units were considered positive.

Sequencing of RT-PCR NTS was performed using an in house designed amplicon-based approach as described before.[3-5] Several modifications were made to the protocol as primer concentrations were increased from 0.125 to 1 pmol for specific primer pairs. In addition modified primers were added to the original primer set to match current circulating variants. Primer sequences and concentrations are available upon request. AMPure XP beads

© 2022 de Hoog MLA et al. *JAMA Network Open*.

purification was only performed on clinical samples with an initial Cp value <32. Libraries were generated using native barcode kits from Nanopore SQK-LSK109 (EXP-NBD104, EXP-NBD114 and EXP-NBD196) and sequencing was performed on a R9.4.1 flow cell multiplexing 96 samples per sequence run.

© 2022 de Hoog MLA et al. *JAMA Network Open*.

eTable 1. Detailed Study Sampling Scheme for All Participants Enrolled

**Repeated Screening (Core study only)**				
**Sample**	**Every 4-6 weeks**			
Nose-Throat-Swab	X			
Saliva	X			
**Outbreak study (SARS-CoV-2 (confirmed on NTS at start))**				
**Sample**	**Start**	**Weekly[#]**	**ARI***	**10 days post-outbreak study completion**
Nose-Throat-Swab	X		X	
Saliva	X	X	X	
Feces	X	X		
Dry Blood Spot	X			X
**Outbreak study (SARS-CoV-2 negative)[$]**				
Nose-Throat-Swab	X		X	
Saliva	X		X	
Feces				
Dry Blood Spot	X			X

[#] Until end of outbreak study

* Only for household member with Acute Respiratory Illness (ARI)

[$]SARS-CoV-2 negative outbreaks were only initiated during the core study

© 2022 de Hoog MLA et al. *JAMA Network Open*.

eTable 2. Proportion of Sample Completeness of SARS-CoV-2–Positive and SARS-CoV-2–Negative Respiratory Outbreaks

**Outbreak (Confirmed SARS-CoV-2)**										
		**NTS; %**	**Saliva; %**			**Feces; %**			**Serology; %**	
Age (years)	Total in outbreak; n	Start	Start	Week 1	Week 2	Start	Week 1	Week 2	Start	End
<12	89	94.4	93.4	51.7	52.8	50.6	50.6	48.3	83.1	50.6
12-17	37	97.3	97.3	59.5	67.6	62.2	54.1	48.6	91.9	75.7
≥18	126	96.0	93.7	56.3	57.1	57.1	50.0	50.8	94.4	64.3
All ages	252	95.6	94.0	55.2	57.1	55.6	50.8	49.6	90.1	61.1
**Outbreak (SARS-CoV-2 negative)***										
<12	208	98.6	96.2	-	-	-	-	-	60.1	37.5
12-17	23	100	100	-	-	-	-	-	95.7	69.6
≥18	244	98.8	96.3	-	-	-	-	-	82.8	57.0
All ages	475	98.7	96.4	-	-	-	-	-	73.5	49.1

*SARS-CoV-2 negative outbreaks were only initiated during the core study

© 2022 de Hoog MLA et al. *JAMA Network Open*.

eFigure 1. Most Common Symptom Combinations per Episode of SARS-CoV-2–Positive and SARS-CoV-2–Negative Respiratory Illness Episodes in Children (Aged <18 Years) and Adults

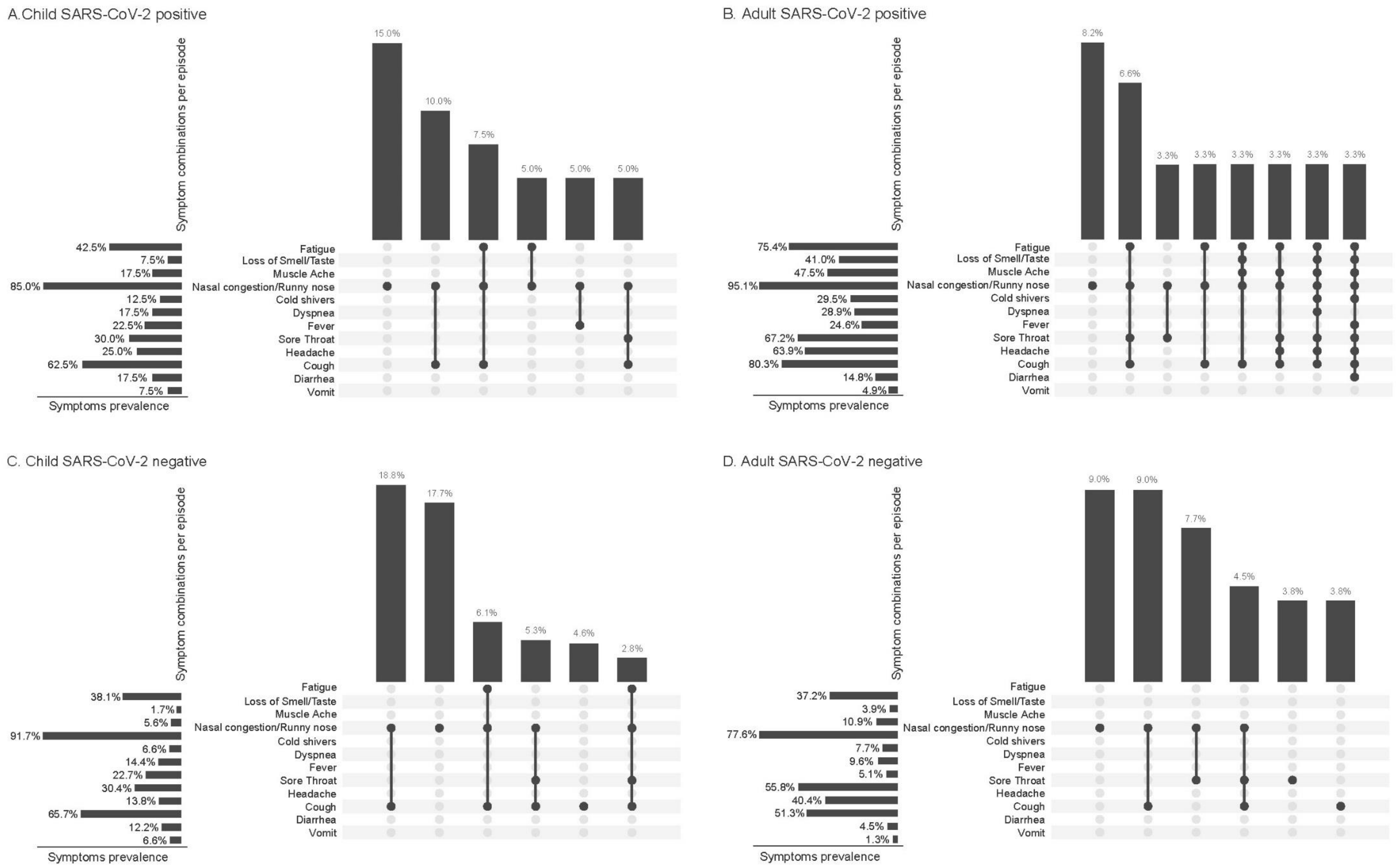

© 2022 de Hoog MLA et al. *JAMA Network Open*.

eFigure 2. Symptom Severity Score Since Onset of ARI Symptoms for SARS-CoV-2–Positive and SARS-CoV-2–Negative Respiratory Illness Episodes

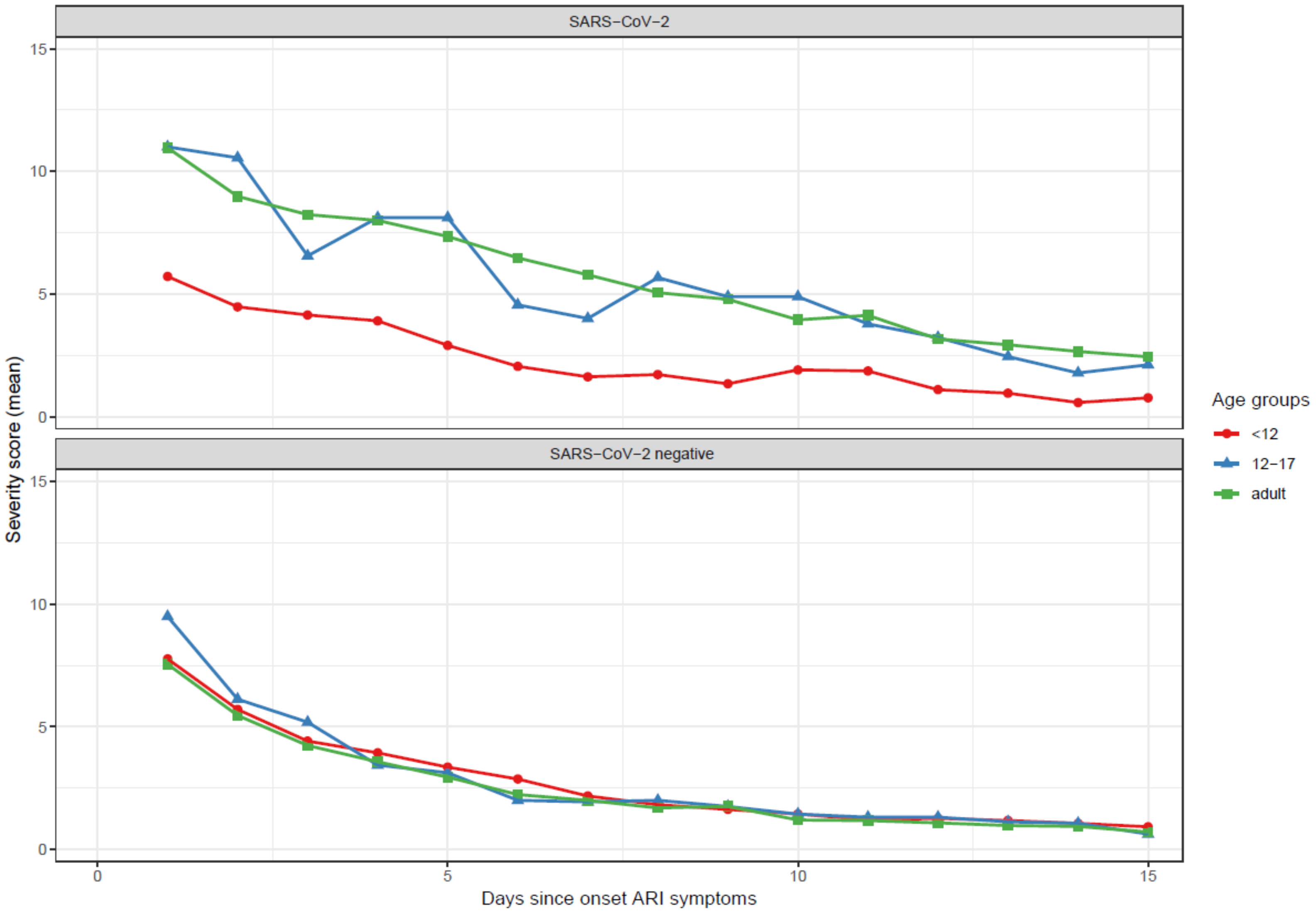

© 2022 de Hoog MLA et al. *JAMA Network Open*.

eReferences

1. Corman VM, Landt O, Kaiser M, Molenkamp R, Meijer A, Chu DKW, et al. Detection of 2019 novel coronavirus (2019-nCoV) by real-time RT-PCR. Eurosurveillance 2020;25. https://doi.org/10.2807/1560-7917.ES.2020.25.3.2000045.
2. van Tol S, Mögling R, Li W, Godeke GJ, Swart A, Bergmans B, et al. Accurate serology for SARS-CoV-2 and common human coronaviruses using a multiplex approach. Emerging Microbes and Infections 2020;9:1965–73. https://doi.org/10.1080/22221751.2020.1813636.
3. Oude Munnink BB, Nieuwenhuijse DF, Stein M, O'Toole Á, Haverkate M, Mollers M, et al. Rapid SARS-CoV-2 whole-genome sequencing and analysis for informed public health decision-making in the Netherlands. Nature Medicine. 2020 Sep 1;26(9):1405–10.
4. Artic Network. https://artic.network/2-protocols.html (accessed Feb 24, 2022).
5. SARS-CoV-2_Sequencing/PCR tiling of COVID-19 virus-minion.pdf at master · CDCgov/SARS-CoV-2_Sequencing · GitHub. https://github.com/CDCgov/SARS-CoV-2_Sequencing/blob/master/protocols/ONT-COVID-19_Tiling/PCR%20tiling%20of%20COVID-19%20virus-minion.pdf (accessed Feb 24, 2022).

© 2022 de Hoog MLA et al. *JAMA Network Open*.
